# Conspiratorial Beliefs and Cognitive Styles: An Integrated Look on Analytic Thinking, Critical Thinking, and Scientific Reasoning in Relation to (Dis)trust in Conspiracy Theories

**DOI:** 10.3389/fpsyg.2021.736838

**Published:** 2021-10-12

**Authors:** Biljana Gjoneska

**Affiliations:** Macedonian Academy of Sciences and Arts, Skopje, North Macedonia

**Keywords:** conspiracy theories, conspiratorial beliefs, cognitive styles, analytic thinking, critical thinking, scientific reasoning

## Abstract

The tendency to believe in conspiracy theories (implying secret and malevolent plots by scheming groups or individuals), incites growing decennial interest among psychological researchers (exploring the associated personality traits, worldviews and cognitive styles of people). The link between the conspiratorial beliefs and the cognitive styles remains of particular interest to scholars, requiring integrated theoretical considerations. This perspective article will focus on the relationship between the propensity to (dis)trust conspiracy theories and three cognitive styles: analytic thinking, critical thinking, and scientific reasoning. Analytic thinking (inclination toward slow and deliberate processing of information in a conscious effort to mitigate biases and reach objective understanding of facts), is a well-studied concept in the context of conspiratorial beliefs, while the negative mutual relationship seems well-evidenced. On the other hand, the evidence on the link with the critical thinking (readiness to consider, reason, appraise, review, and interpret facts to update existing beliefs) has only started to emerge in the last years. Finally, scientific reasoning (ability to apply principles of scientific inquiry to formulate, test, revise and update knowledge in accordance with new evidence), is the least studied of the three cognitive styles in relation to conspiracy theories. The present article will: (a) revise the (lack of) *scientific consensus* on the definitional and conceptual aspects (by providing theoretical framework); (b) summarize the *state of the art* on the subject (by providing overview of empirical evidence); (c) discuss *directions for future research* (especially in relation to the COVID-19 pandemic). An integrated perspective on the relationship between conspiratorial beliefs and cognitive styles of people, may serve to inspire future behavioral interventions.

## 1. Introduction

One of the powerful academic portrayals of a world filled with conspiracies, depicts an inhospitable environment, dominated by “*a gigantic and yet subtle machinery of influence set in motion to undermine and destroy a way of life*” (Hofstadter, 1964, p. 29). This portrait however, pertains less to the external world, and more to some internal worldviews. Hence, it is not a depiction, but a reflection of sorts, offering a glimpse into the mental states of people with pronounced tendency to endorse conspiratorial narratives as explanations for important events, and cultivate persistent beliefs that powerful others are secretly plotting to harm them (Hofstadter, 1964; Moscovici, 1987; Goertzel, 1994; Swami et al., 2011; Bruder et al., 2013; Wood and Douglas, 2015; van der Linden et al., 2021).

Interestingly, conspiratorial narratives are often regarded as both a most probable scenario (by people who subscribe to conspiratorial beliefs) and a least probable account of events (by others). In a similar fashion, people who are inclined toward conspiratorial thinking might believe to be “critical freethinkers” themselves (Lantian et al., 2021), while being regarded as gullible by others (van Prooijen, 2019). Psychological researchers have intensified their effort to understand the complexities of these radically opposing perceptions, and in doing so have amassed an impressive body of knowledge on personality traits, cognitive styles and worldviews that are frequently associated with beliefs in conspiracy theories (for an overview see Douglas et al., 2017; Goreis and Voracek, 2019; Lantian et al., 2020). However, the topic remains complex, multilayered and intricate, with real-life implications for individuals, groups and whole societies. This has been especially evident in the time of the COVID-19 pandemic during which the so-called “contagious conspiracism” has been a prominent feature of the global cultural landscape (Sturm and Albrecht, 2021) and has negatively affected the health of many citizens worldwide (Freeman et al., 2020; Jolley and Paterson, 2020; Marinthe et al., 2020). To adequately tackle the problem, it is the belief of this author that a joint effort by experts in several psychological disciplines (including social, political, educational, personality, and cognitive psychology) is required. Thus far, cognitive and educational psychologists together with philosophers have mainly focused on postulating conceptual frameworks of cognition and rationality (Stanovich and Stanovich, 2010; Díaz et al., 2021), while social and political psychologists have mainly directed their effort toward experimental investigations of the conspiratorial beliefs.

The present article outlines a unified perspective on susceptibility to (dis)trust conspiracy theories, in relation to three distinctive cognitive styles: analytic thinking, critical thinking and scientific reasoning. Specifically, the study addresses three crucial questions:

1. What is the appropriate scientific model to use when researching the three cognitive styles, considered in reference to the psychological research on conspiratorial beliefs? An *integrated theoretical framework* (with clear and delineated definitions), will be introduced in response to this question. This is as a novel perspective on the explored subject matter.
2. What are some of the most important contributions in psychological literature on the link between the conspiratorial beliefs and the three cognitive styles? A *broad overview* of existing evidence (highlighting most important findings), will be offered in response to this question.
3. What is the potential for applying findings from psychological research on conspiracy theories to benefit our daily lives? In the concluding section, *existing methodology and potential implications* will be discussed, with hopes they will serve to inspire future behavioral interventions and inform public policies.

Henceforth, the term “conspiratorial beliefs” (Goertzel, 1994) will be used as an umbrella for other labels that are frequently utilized in psychological literature on conspiracy theories including: “conspiracist ideation” (Swami et al., 2011), “conspiracy mentality” (Bruder et al., 2013), “conspiratorial mindset” (Moscovici, 1987; van der Linden et al., 2021), or “conspiratorial worldview” (Wood and Douglas, 2015). Therefore, the term will imply a “monological belief system,” Goertzel (1994) marked by a general propensity to believe in conspiracy theories, rather than a content-specific belief (Sternisko et al., 2020) in a particular conspiracy theory (Sutton and Douglas, 2020).

The term “cognitive styles” is also used in a variety of related contexts within psychological research on conspiracy theories (Georgiou et al., 2019; Ballová Mikušková and Čavojová, 2020; Lantian et al., 2020). The basic description however, is borrowed from a comprehensive review of psychological studies on cognitive styles (Kozhevnikov, 2007) to outline “a psychological dimension representing consistencies in an individual's manner of cognitive functioning” (ibid, p. 464). As such, cognitive styles are relatively stable, partly fixed and innate. However, they are not entirely “inborn structures, dependent only on an individual's internal characteristics, but, rather, are interactive constructs that develop in response to social, educational, professional, and other environmental requirements” (ibid, p. 477). Hence, they are “complex, multifaceted psychological variables that affect the way a person prefers to process information” and refer “to the way people solve problems, make decisions and undertake tasks” (Peterson et al., 2009, p. 521). In the present article the label will be used in reference to *analytic thinking, critical thinking and scientific reasoning*.

Each of the three cognitive styles is guided by rationality and goals for reliable *information processing, decision making, and problem solving*, and they all rely on *thinking dispositions, metacognitive strategies, and advanced cognitive skills* (Halpern, 1998; Dunbar and Fugelsang, 2005; Ku and Ho, 2010). The dispositional tendencies direct the execution of tasks, metacognitive strategies regulate execution of tasks, while advanced cognitive skills enable acquisition, retention and transfer of knowledge from executed tasks (Ku and Ho, 2010). In this regard, metacognitive strategies and advanced cognitive skills are highly reminiscent of the term “mindware” that is used in reference to “rules, knowledge, procedures, and strategies” that can be retrieved from memory to assist in decision making and problem solving processes (Stanovich and Stanovich, 2010, p. 215).

## 2. Analytic Thinking, Critical Thinking, and Scientific Reasoning: An Integrated Theoretical Framework

First, let us focus on the three cognitive styles, by providing definitions of terms, clear descriptions of their meanings, and delineation of mutual relationships.

- *Analytic thinking* predominantly implies proneness to engage in a slow, controlled and deliberate processing of information. The thinking disposition is engaged to mitigate biases and establish reliable *understanding of facts* (Sloman, 1996; Kahneman and Frederick, 2002; Kozhevnikov, 2007; Franssens and De Neys, 2009; Kahneman, 2011).
- *Critical thinking* implies readiness and willingness to (re)consider, reason, (re)appraise, review and interpret facts, in order to facilitate good judgment, and secure reliable updating of beliefs (Lai, 2011). It contains the three components (Halpern, 1998), but is probably most reliant on the *disposition toward analytic thinking* and the *metacognitive strategies for repeated engagement in analytic thinking*. This might be the reason why critical thinking is described as “a self-directed, self-disciplined, self-monitored, and self-corrective thinking” (Elder and Paul, 2000, p. 29).
- *Scientific reasoning* also comprises of the three components, but is especially dependent on the *advanced cognitive skills*. It includes induction, deduction, analogy, causal reasoning, and other competencies which are employed for the purposes of scientific inquiry and problem solving during the critical thinking processes. They help people formulate, test, and revise hypotheses to solve problems, as a way to integrate new evidence into their existing *system of knowledge* (Dunbar and Fugelsang, 2005; Han, 2013; Díaz et al., 2021).

The proposed theoretical framework is informed by existing social psychological research, and conceptualized in consultation with related literature from cognitive psychology, educational psychology and philosophy (see corresponding references above). Most notably, the proposed tripartite model of cognitive styles (including analytic thinking, critical thinking and scientific reasoning), could be considered as complementary to the existing tripartite model of the mind (including the autonomous, algorithmic and reflective mind) by Stanovich and Stanovich (2010). Specifically, the three cognitive styles rise above the basic cognitive abilities as “microstrategies for cognitive operations” (Stanovich and Stanovich, 2010, p. 215), since they are driven by goals, beliefs and general knowledge. Therefore, the three cognitive styles are also related to the concept of rationality, providing an upgraded and fine-grained perspective on the properties of the reflective mind.

**Figure 1:**
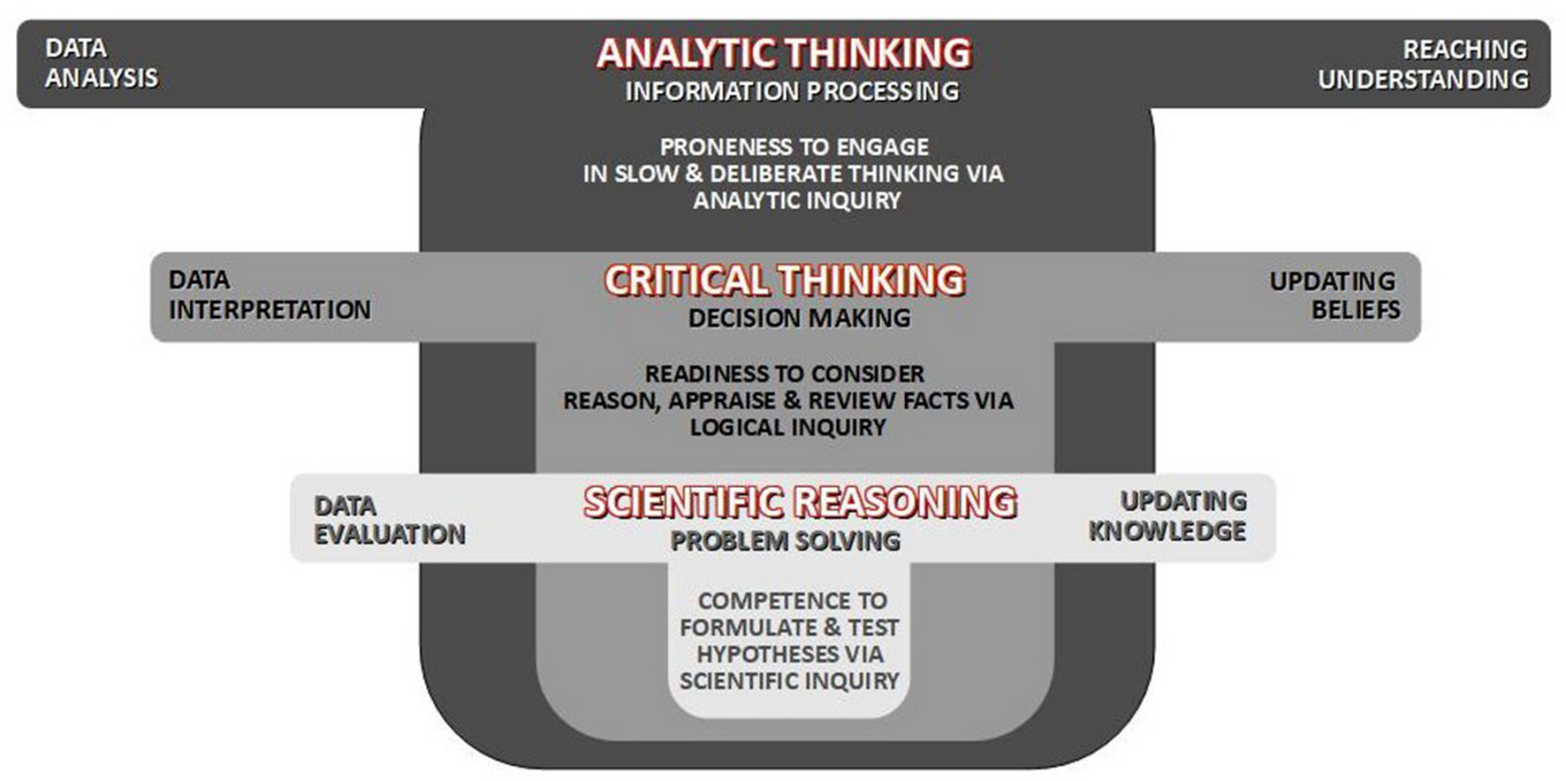
Conceptual framework of cognitive styles: analytic thinking (the broadest and lowest in order), critical thinking, and scientific reasoning (the narrowest and highest in order) are conceptualized as related and nestled constructs.

In addition to advancing the existing model by Stanovich and Stanovich (2010), the proposed framework is useful in shedding light on the hierarchical organization of cognitive styles and their hypothetical relationships (Figure 1). Namely, the three cognitive styles can be represented within a nested structure, with analytic thinking considered as a lowest-order and broadest construct (comprising of most general set of dispositions, metacognitive strategies and advanced skills), while the scientific reasoning considered as a narrowest and highest-order construct (comprising a most specialized subset of the three).

Specifically, analytic thinking and critical thinking can be considered as neighboring but distinct cognitive styles (Lantian et al., 2021), with the former usually referred as a broader set of the latter. In critical thinking, the general tendency for slow, deliberate, explicit (Kahneman and Frederick, 2002; Kahneman, 2011), detail-oriented (Kozhevnikov, 2007) and resource-demanding analysis (Franssens and De Neys, 2009), is coupled with more elaborate dispositions and metacognitive strategies, sometimes referred as a “mindware” (Stanovich and Stanovich, 2010). These include the critical thinking dispositions for persistent, honest, clear, caring and concerned pursuit of the truth (Ennis, 1996), the instrumental rationality (as motivation to achieve one's goals) and the epistemic rationality (as motivation to endorse evidence-based beliefs, but refrain from beliefs that are unfounded) (Kelly, 2003).

In this framework, and as seen on Figure 1, critical thinking and scientific reasoning can also be considered as related yet separate constructs, with the latter understood as a subset of the former (Dowd et al., 2018). Broadly speaking, the acts of *thinking* and *reasoning* differ in that thinking involves more general cognitive processes for systemic transformation of mental representation of knowledge, while reasoning includes specialized cognitive processes aimed at drawing conclusions from initial premises (Holyoak and Morrison, 2005; Díaz et al., 2021). More specifically, the acts of *critical thinking* and *scientific reasoning* also differ from each other. The former is related to *interpretation of facts, updating of beliefs, making sound judgments* and *delivering reliable decisions*. On the other hand, scientific reasoning is related to *evaluation of facts, updating of knowledge and problem solving strategies*. Overall, critical thinking is grounded in principles of logical inquiry, while scientific reasoning in scientific principles and methods (Zimmerman, 2007).

Scientific reasoning in particular, encompasses a specialized subset of advanced cognitive abilities, metacognitive strategies and thinking dispositions that permeate the field of science, and include (but may not be limited to) the following operations: exploration of a problem (i.e., identification of main variables and their mutual relationships via inductive and deductive reasoning), generation of hypotheses (i.e., concept formation, formulation of premises and expected outcomes), hypothesis testing (i.e., isolation, controlling and manipulation of variables via experimentation), and evaluation of consequences (Dunbar and Fugelsang, 2005; Han, 2013). Scientific reasoning is important for individuals, because it improves their ability to formulate, test, revise and update knowledge. The societal benefits are evident across all levels of education, career opportunities and daily social interactions that require problem solving competencies (Han, 2013). Nonetheless, its relationship with conspiratorial beliefs remains scarcely explored (as explained in the following section).

## 3. Cognitive Styles and Conspiratorial Beliefs: An Overview of Empirical Evidence

Next, let us consider the link between the cognitive styles described in section 2 and conspiratorial beliefs. All three styles are essential for reliable interpretation of events, and making sense of one's environment. Broadly speaking, analytic thinking helps us to discern a truth from a lie or a fact from a fiction (in everyday processing of information), critical thinking helps us to decide whether to believe or not an (un)reliable information (when making decisions and judgments), while scientific reasoning helps us to gain wholesome understanding of the observed subject matter (by solving problems and finding solutions). A failure in any of these domains might be associated with increased conspiracism, because it is a signal of “crippled epistemology” (Vermeule and Sunstein, 2009). This has already been evidenced in literature on flawed heuristics, cognitive biases and logical fallacies. The prominent examples include the tendency to perceive illusory patterns (Prooijen et al., 2018; van der Wal et al., 2018), the illusion of explanatory depth (Vitriol and Marsh, 2018), and the proneness toward conjunction fallacy (Brotherton and French, 2014), all of which have been associated with conspiratorial beliefs. On a more complex level, people with pronounced propensity toward conspiracy theories, also exhibited a tendency to endorse belief systems that are epistemically unsubstantiated. These include supernatural, superstitious, spiritualistic, paranormal, pseudo-scientific beliefs, paranoid and schizotypal ideations (Hofstadter, 1964; Darwin et al., 2011; Barron et al., 2014; Lobato et al., 2014; Georgiou et al., 2019; van Prooijen, 2019).

The (bi)directionality and the causality of these relationships is still unclear, given the limitations of the conducted studies (as explained in the discussion). On one hand, it seems plausible to assume that flawed heuristics and faulty reasoning, would result with increased tendency to believe in conspiratorial narratives. In this case, many strategies to improve analytic, critical and scientific thinking or reasoning, would serve to protect from such beliefs by enhancing observation, examination, checking, and rejection of unwarranted claims. On the other hand, the reverse causality also seems possible, where pronounced (pre)disposition toward conspiratorial beliefs, negatively affects information processing, decision making and problem solving, thus leading to faulty reasoning and flawed beliefs or knowledge systems.

We highlight findings that support the notion that analytic thinking reduces the tendency to engage in overly religious, paranormal (Pennycook et al., 2012) and conspiratorial beliefs (Swami et al., 2014). Overall, the link between the analytic thinking and the conspiratorial beliefs is negative, well-evidenced and robust (Ståhl and van Prooijen, 2018; van der Wal et al., 2018; Georgiou et al., 2019; Wagner-Egger et al., 2019).

A number of studies have gone further, analyzing the link between conspiratorial beliefs and: (a) beliefs about the nature of knowledge i.e., epistemic beliefs (Garrett and Weeks, 2017); (b) open-minded beliefs about the importance of evidence (Pennycook et al., 2020a); and (c) motivation to endorse beliefs that are calibrated with evidence i.e., epistemic rationality (Ståhl and van Prooijen, 2018; Adam-Troian et al., 2019). Research on epistemic rationality, in particular, has shown that it moderates the relationship between conspiratorial beliefs and lower-level constructs in the following way: (a) it strengthens the negative relationship with general cognitive abilities (Adam-Troian et al., 2019); and also (b) it strengthens the negative relationship with analytic thinking (Ståhl and van Prooijen, 2018). Overall, these studies have highlighted the pivotal role of the so-called “mindware” and various metacognitive strategies for the enhanced resistance toward conspiratorial narratives. In a recent study, Lantian et al. (2021), the authors utilized Ennis-Weir critical thinking essay test (Ennis, 1996) and the generic conspiracist beliefs scale (Brotherton et al., 2013) to directly test the link, concluding that “conspiracy believers have less developed critical thinking ability.”

Lastly, research on the relationship between conspiracy theories and scientific reasoning (usually assessed via the scientific reasoning scale) Drummond and Fischhoff (2017) is still scarce. In fact, it remains limited to a single research group, reporting several findings over the last few years and confirming the negative correlation between this cognitive style and susceptibility toward cognitive biases (Čavojová and Brezina, 2019) or COVID-19 related conspiratorial beliefs (Čavojová and Brezina, 2019; Čavojová et al., 2020).

## 4. Discussing Implications and Future Perspectives

The framework proposed (in section 2) integrates theoretical considerations on three distinct cognitive styles (analytic thinking, critical thinking and scientific reasoning), into an organized system (with concise definitions of terms, clear description of meanings, and delineated mutual relationships). It is consistent with past psychological research, while at the same time providing fresh insights on the following aspects:

- The constructs: they can be thought of as nested within each other, with analytic thinking comprising the broadest set, and scientific reasoning as the narrowest and most specialized subset.
- The heuristics: analytic thinking relies on the dispositions for slow and conscious processing of information, critical thinking on the dispositions and the metacognitive strategies for reliable decision making, while scientific reasoning on the advanced cognitive skills and competencies for problem solving.
- The goals: analytic thinking is oriented toward unbiased and objective understanding of facts in daily situations, critical thinking toward reliable update of beliefs, while scientific reasoning toward updates of knowledge systems.

The overview on past research (in section 3) has revealed that the investigations have been: partial (because they explored the link between conspiratorial beliefs and separate cognitive styles in separate research contexts), sporadic (especially with regards to the research on the link with the critical thinking), or even accidental (especially with regards to the research on the link with the scientific reasoning). Furthermore, the investigations were predominantly cross-sectional and correlational, and therefore with limited ability to make conclusions on the causal inference. In addition, there has been little progress in standardizing methodology and empirical approaches across studies. For instance, most analyses in this area rely on self-reported measures (i.e., scales and questionnaires), and rarely on experimental designs (e.g., studies on cognitive biases and logical fallacies). While most of the studies employed quantitative analyses for assessment of results, the measurement of the variables (e.g., the conspiratorial beliefs) has been conducted on differing scales, and some of the scales had unknown psychometric properties.

Integrated theoretical considerations can serve as basis for a unified approach in empirical studies. Specifically, they can shape future psychological research to: (a) build models that will account for all presented variables; (b) conduct experiments with ecologic validity preferably outside of laboratory settings; (c) perform complex statistical analyses (e.g., hierarchical regressions and structural equation modeling) that explore mutual relations between all proposed variables and test the overall model. More realistic models and improved experimental designs can inspire future behavioral interventions in the fight against misinformation and conspiracy theories, by cultivating the capacity for analytic, critical and scientific thinking (van der Linden et al., 2020; Lewandowsky et al., 2021). This is especially relevant in the context of the COVID-19 pandemic where millions across the globe are inundated by conspiracy theories that have been linked to engagement in non-normative prevention behaviors (Marinthe et al., 2020), decreased trust in government and lack of compliance with official public health recommendations (Freeman et al., 2020), or even engagement in risky and violent behaviors (Jolley and Paterson, 2020).

Emerging evidence regarding pandemic-related conspiratorial beliefs and various cognitive markers, suggests that: (a) they are positively linked with a group of cognitive biases, marked by an increased tendency to make premature conclusions (delivered on basis of low subjective probability estimates, lack of sufficient evidence, or even in the face of disconfirmatory evidence) (Kuhn et al., 2021); (b) they are negatively linked with scientific reasoning (Čavojová et al., 2020); and most importantly (c) they can be reduced by nudging individuals to consider accuracy of presented statements (Pennycook et al., 2020b). In this respect, strategies that aim to enhance rationality seem to have potential in reducing prevalence of conspiratorial beliefs. For example, asking participants to judge the accuracy of a piece of information (in order to secure more reliable analysis and enhanced analytical thinking), or to judge subjective importance of an information (in order to secure more accurate interpretation and enhance critical thinking), or just providing digital literacy tips (for improved scientific reasoning) have been shown to reduce the spread of COVID-19 misinformation (Epstein et al., 2021).

## 5. Conclusions

The present article *offers a perspective* on the current scientific consensus, and *opens a perspective* toward future investigations of the link between the conspiratorial beliefs and three cognitive styles: analytic thinking, critical thinking and scientific reasoning. It does so, by outlining a clear *perspective on others'* works, and conceptualizing the *author's perspective* in an integrated theoretical framework. The literature overview is given in a condensed format, to serve as a basis for future systematic reviews or meta-analyses. Also, the theoretical framework is quite broad, and could be further advanced in a study focused exclusively on theoretical improvements and hypothesis development. This study will hopefully inspire a dialogue between researchers from different disciplines seeking to develop unified and multidisciplinary approach in the fight against misinformation and conspiracy theories.

## Data Availability Statement

The original contributions presented in the study are included in the article, further inquiries can be directed to the corresponding author.

## Author Contributions

BG has conceptualized the study, drafted the manuscript, and thoroughly revised the contents before submitting. The style and language were checked by native English speaker.

## Conflict of Interest

The author declares that the research was conducted in the absence of any commercial or financial relationships that could be construed as a potential conflict of interest.

## Publisher's Note

All claims expressed in this article are solely those of the authors and do not necessarily represent those of their affiliated organizations, or those of the publisher, the editors and the reviewers. Any product that may be evaluated in this article, or claim that may be made by its manufacturer, is not guaranteed or endorsed by the publisher.
